# Association of morphine vs. fentanyl prescription dispensation with adverse clinical outcomes

**DOI:** 10.3389/fphar.2025.1579634

**Published:** 2025-06-25

**Authors:** Carlen Reyes, Maria Antonia Pou, Cesar Diaz-Torne, Cristina Carbonell-Abella, Carlos Brotons, Daniel Martinez-Laguna

**Affiliations:** ^1^ Sardenya Primary Health Care Research Center-Research Institute Sant Pau (EAP Sardenya-IR Sant Pau), Barcelona, Spain; ^2^ Sistema d’Informació per al Desenvolupament de la Investigació en Atenció Primària, Fundació Institut Universitari per a la recerca a l’Atenció Primària de Salut Jordi Gol i Gurina (IDIAPJGol), Mataró, Spain; ^3^ Primary Care, Catalan Health Institute (ICS), Barcelona, Spain; ^4^ Rheumatology Department, Hospital de la Santa Creu i Sant Pau, Barcelona, Spain; ^5^ Autonomous University of Barcelona, Barcelona, Spain

**Keywords:** primary healthcare, fentanyl, morphine, cohort study, Spain

## Abstract

**Introduction:**

The aim was to assess the associations between morphine, fentanyl and adverse events in primary care patients.

**Methods:**

A retrospective, propensity-score-weighted cohort study using a primary-care database covering >75% population of Catalonia, Spain was conducted. Patients aged ≥18 years with ≥1 year of available data and incident dispensation of morphine or fentanyl, were included from 1st January 2007 to 31st December 2017. Outcomes were all-cause mortality, cardiac arrhythmias, fractures (hip, pelvis, vertebra, wrist, humerus), constipation, delirium, falls, opioid abuse/dependence, and sleep disorders while on treatment. Risk ratios (RRs) and hazard ratios (HRs) with 95% confidence intervals (CIs) were calculated using cause-specific Cox models.

**Results:**

A total of 12,632 patients (3,040 with morphine and 9,695 with fentanyl) were included (median [IQR] age, 78.4 [63.8; 86.1] years; 63.6% female). Compared with morphine, fentanyl dispensation was associated with a higher risk of fractures (incidence: 6.92 vs. 4.13 per 1,000 dispensations-month; HR, 1.63 [95% CI, 1.15–2.32]; RR, 1.78 [95% CI, 1.25–2.53]), especially in men and in those <65 and over >80 years old. No difference was observed for the rest of outcomes.

**Conclusion:**

Among outpatients, a new prescription dispensation of fentanyl, compared with morphine, was associated with a higher risk of fractures. The findings should be interpreted cautiously given the potential for residual confounding.

## 1 Introduction

Fentanyl and morphine are long-acting opioids indicated for the management of severe pain (Alorfi, 2023). Their increased prescription over the past decade has contributed to the opioid epidemic reported in the United States. In 2021, the age-adjusted drug overdose death rate driven by fentanyl rose to 22 per 100,000 standard population (Spencer et al., 2022) and the number of emergency visits involving fentanyl products totaled 123,563 visits (Drug Abuse Warning Network DAWN, 2021). In Europe, the overall burden of long-acting prescriptions remains lower than that reported in the United States (Pierce et al., 2021). Until 2017, prescriptions of fentanyl and morphine for non-cancer indications increased in primary care settings in both Spain and Germany (Xie et al., 2022; Hurtado et al., 2020; González-Bermejo et al., 2021; Rosner et al., 2019). The subsequent stabilization observed in Spain, based on the analysis of Defined Daily Doses (DDD) (Agencia Española de Medicamentos y Productos Sanitarios, 2024), contrasts with findings from other observational studies that reported a continued rise in the number of prescription dispensations during the same period, suggesting a potential underestimation of actual use.

Both fentanyl and morphine have similar indications for non-cancer pain, and they have been shown to provide similar benefits for pain relief and physical functioning (Fleischman et al., 2010). Although the comparative safety between these opioids has been explored among patients with cancer or in hospital settings (Clark et al., 2004; Hu et al., 2021), with a lower risk of adverse events observed among fentanyl users (Hu et al., 2021; Manirakiza et al., 2020) head-to head comparisons among outpatient populations are limited and show inconsistent results (Domínguez-Berjón et al., 2008; Hartung et al., 2007; Le et al., 2023). While a large cohort study conducted in the United States found no significant difference in mortality risk between fentanyl and morphine users’ (Domínguez-Berjón et al., 2008) studies in several European countries, have linked fentanyl use to a higher risk of opioid dependence compared to morphine (Hartung et al., 2007).

Additionally, data from the UK revealed that fentanyl dispensed by community pharmacies was associated with a higher incidence of unintentional deaths (Le et al., 2023). Given the increasing use of fentanyl and morphine in primary care settings to manage severe painful conditions, their comparative safety profiles should be assessed, and their benefits weighed against potential harms. This population-based cohort study compared risks of all-cause mortality, arrhythmias, fractures, constipation, delirium, falls, opioid abuse/dependence, and sleep disorders between patients who received prescriptions for fentanyl versus morphine in a primary care setting.

## 2 Materials and methods

### 2.1 Data sources

This retrospective cohort study used data from the System for the Development of Research in Primary Care (SIDIAP), which comprises routinely collected anonymized electronic primary-care records (ICD-10 codes) and sociodemographic data for a representative sample (>75%) of Catalonia, Spain (Recalde et al., 2022). This database links with the national pharmacy claims for community pharmacy dispensations (Anatomical Therapeutic Chemical (ATC) code, dispensation date and number of packages).

### 2.2 Study design and cohort definition

We conducted a retrospective cohort study to compare the risks of adverse events among patients with new prescription dispensations of fentanyl versus morphine. Morphine was used as the active comparator to mitigate confounding by indication, as both fentanyl and morphine are strong opioids with similar indications and are prescribed in Spain for severe pain (Alorfi, 2023). New dispensation was defined by applying a fixed 12-month look-back period in which the patient had continuous data coverage but did not have any opioid dispensations or non-steroidal anti-inflammatory drugs. If a patient had multiple dispensation episodes, dispensation exposure periods were created by concatenating the dispensations with a maximum gap allowed of 45 days from the date of the last dispensation to the date of the following dispensation. Once these dispensation exposure periods were created, only those that complied with the 12-month look back period were included.

All patients who were dispensed fentanyl or morphine between 1 January 2007, and 31 December 2017 were identified and categorized in the fentanyl or morphine cohort according to the first dispensed drug. Cohorts were constructed by including patients who were aged 18 years or older, had at least 1 year of database enrollment before the first drug dispensation (index date). Patients with any other opioid or NSAID in the 12-month look-back period, those with cancer or previous major surgery (amputation and joint replacement surgery), or any of the studied outcomes on or before the index date were excluded.

### 2.3 Baseline characteristics

The study population was characterized at the index date by considering the following potential confounders: sociodemographic factors (age, sex, and socioeconomic deprivation), medical conditions, and healthcare utilization. Socioeconomic deprivation was measured with the MEDEA (Mortalidad en áreas pequeñas Españolas y Desigualdades Socioeconómicas y Ambientales) index that describes socioeconomic and environmental inequalities among 175 small areas of Spain. This index is divided into equally sized quintiles with the first quintile representing the least deprived and the last quintile representing the most socioeconomically deprived (Domínguez-Berjón et al., 2008). Healthcare utilization was quantified by the frequency of general practice visits in the past 12 months.

The following acute and chronic health conditions expected to be linked to opioid prescription dispensation or associated with any of the study outcomes were identified. These included pulmonary edema, diarrhea, chronic cough, migraine, burn injuries, cardiovascular events, peripheral vascular disease, diabetes (type 1 and type 2), malabsorption disorders, chronic obstructive pulmonary disease, chronic musculoskeletal pain disorders, rheumatological disorders, Alzheimer’s disease, Parkinson’s disease, chronic liver and chronic kidney disease.

### 2.4 Study outcomes

The study outcomes were cardiac arrhythmia, delirium, fractures (hip, pelvis, wrist, humerus, and vertebra), falls, sleep disorders (sleep apnea, somnolence), constipation, opioid dependence/abuse and all-cause mortality while on treatment with fentanyl or morphine. The ICD-10 codes used to identify the study outcomes are reported in Supplementary Table S1.

### 2.5 Statistical analyses

Study variables were described in tables by type of exposure drug (morphine vs. fentanyl). Categorical variables were described by the frequency and percentage of each category. Continuous variables were described by the mean and standard deviation or by the median and interquartile range, depending on the distribution of the variables. Unadjusted incidence rates and rates for 1,000 dispensation-month for each of the events of interest stratified by type of drug exposure were calculated.

To compare the incidence of the adverse events between morphine and fentanyl exposure periods a log-binomial regression was estimated with propensity score inverse probability weighting. Risks ratios were estimated and reported. To compare the time to first adverse event between morphine and fentanyl exposition periods Cox survival models were estimated with propensity score inverse probability weighting. Hazard ratios were estimated and reported.

The propensity score was used to balance the two exposure cohorts based on observed confounding variables. The propensity score represents the probability of having a morphine or fentanyl exposure period, conditional on the values of observed confounding variables. In these analyses, the propensity score was calculated using Bayesian additive regression trees. Variables included were age, sex and the chronic and acute clinical confounders.

Sensitivity analyses were carried out to assess the association between fentanyl dispensations, compared to morphine, and the different outcomes, stratified by sex and age (<65, 65–80 and >80 years old).

The conditions of use of the models were validated and, whenever possible, 95% confidence intervals were calculated. Significance levels were set at the 5% level. All analyses were performed using the statistical program R version 4.4.0 (2024-04-24) for Windows.

### 2.6 Institutional review board statement

The local ethics committee (“Comitè Ètic d'Investigació amb medicaments” (CEIm)) of the “Fundació Institut Universitari per a la recerca a l’Atenció Primària de Salut Jordi Gol i Gurina (IDIAPJGol))” approved this study with registration number P18/085. Informed consent was not required.

### 2.7 Patient and public involvement

This study used routinely collected health data. No patients were involved in the design, conduct, reporting, or dissemination plans of our research.

## 3 Results

### 3.1 Study population and baseline characteristics

**FIGURE 1:**
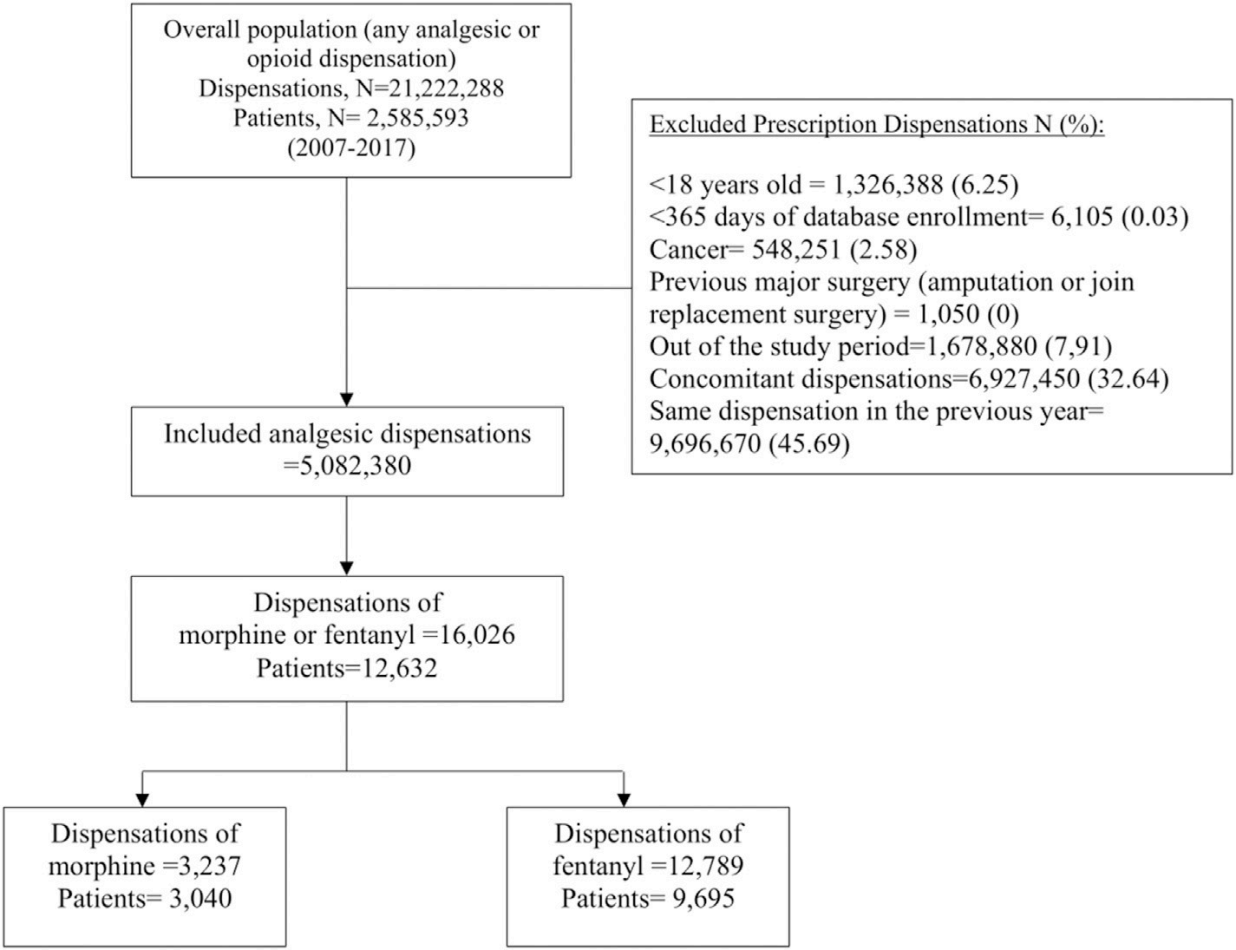
Flow chart of the included and excluded prescription dispensations of fentanyl and morphine during the study period 2007–2017.

A total of 12,632 patients with a dispensation of fentanyl or morphine during the study period were initially identified and followed for 90,448.41 person-year for fentanyl and 24,776.10 person-year for morphine. Of these, 3,040 (24.1%) had a prescription dispensation of morphine and 9,695 (76.8%) had a prescription dispensation of fentanyl. Overall, 103 patients (<1%) contributed to more than 1 exposure period of the study drugs. Figure 1 shows the participant selection process.

**FIGURE 2:**
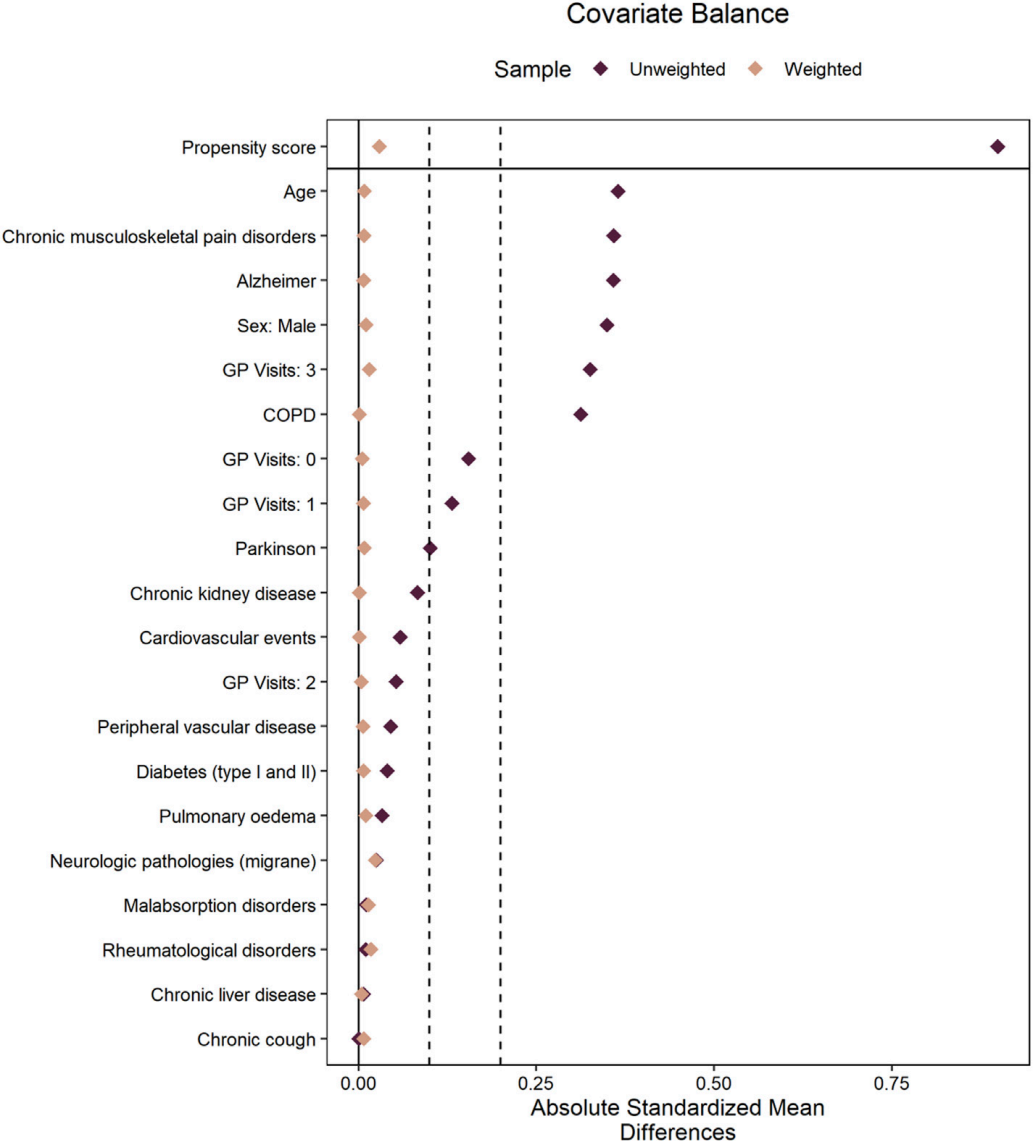
Love plot of the propensity score weighting to balance the morphine and fentanyl exposure cohorts based on observed confounding variables. Vertical dashed lines have been added at 0.1 and 0.2 values. Absolute SMD values below 0.1 are often considered to indicate a good balance, while 0.2 and above indicate an imbalance that requires further investigation.

Table 1 reports the participants’ baseline characteristics before propensity score weighting. Figure 2 shows the love plot of the population after weighting, with the two cohorts of new users comparable across all observed features.

**TABLE 1:** Baseline characteristics of the morphine and fentanyl users (before weighting).

Variables		Morphine, N = 3,040	Fentanyl, N = 9,695
Sex	Female, N (%)	1,629 (53.6)	6,464 (66.7)
	Male, N (%)	1,411 (46.4)	3,231 (33.3)
Age (first dispensation), median [IQR]		83.2 [71.3; 89.8]	76.8 [61.6; 84.8]
BMI (first dispensation), median [IQR]		26.2 [23.1; 29.5]	28.0 [24.6; 31.7]
Socio-economic status MEDEA index^1^, N (%)			
U1		437 (27.8)	1,261 (21.4)
U2		312 (19.8)	1,261 (21.4)
U3		315 (20.0)	1,208 (20.5)
U4		276 (17.6)	1,130 (19.2)
U5		233 (14.8)	1,029 (17.5)
Clinical conditions, N (%)			
Pulmonary oedema		3 (0.1)	2 (0.0)
Diarrhea		0 (0.0)	0 (0.0)
Chronic cough		5 (0.2)	20 (0.2)
Neurologic pathologies (migraine)		0 (0.0)	4 (0.0)
Burn injuries		0 (0.0)	4 (0.0)
Cardiovascular events		48 (1.5)	109 (0.9)
Peripheral vascular disease		173 (5.3)	559 (4.4)
Diabetes (type I and II)		815 (25.2)	2,998 (23.4)
Malabsorption disorders		8 (0.3)	25 (0.2)
Chronic obstructive pulmonary disease		624 (19.3)	1,098 (8.6)
Chronic musculoskeletal pain disorders		1,057 (32.7)	6,401 (50.1)
Rheumatological disorders		13 (0.4)	60 (0.5)
Alzheimer		447 (13.8)	487 (3.8)
Parkinson		159 (4.9)	378 (3.0)
Chronic liver disease		18 (0.6)	78 (0.6)
Chronic kidney disease		536 (16.6)	1,738 (13.6)
General Practitioner visits, N (%)			
0		991 (30.6)	4,852 (37.9)
1		649 (20.1)	3,268 (25.6)
2		410 (12.7)	1,851 (14.5)
3+		1,187 (36.7)	2,818 (22.0)

Patients with a fentanyl dispensation were younger and included a larger proportion of females compared to those with morphine dispensations; median [Interquartile range, IQR] ages were 76.75 [61.58; 84.75] and 83.16 [71.25; 89.75] years, and there were 6,464 (66.7%) and 1,629 (53.6%) females respectively. A higher proportion of patients with fentanyl prescription dispensations was observed among those socioeconomically deprived compared to those dispensed morphine (17.5% of fentanyl patients versus 14.8% of morphine patients in U5).

**FIGURE 3:**
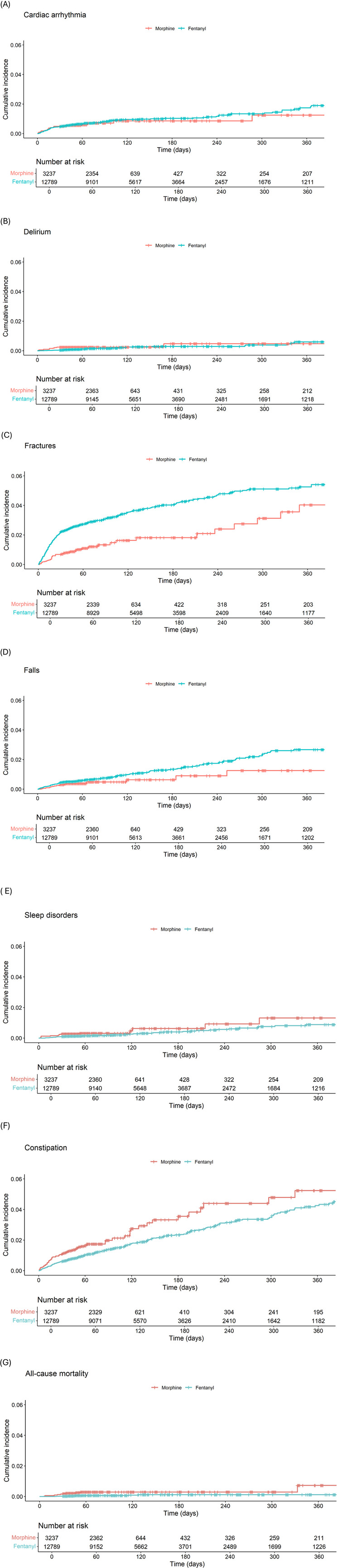
Kalpan Meier plots showing the cumulative incidence and numbers at risk in each morphine and fentanyl cohort for **(A)** Cardiac arrhythmia, **(B)** Delirium, **(C)** Fractures (hip, pelvis, vertebra, wrist, humerus), **(D)** Falls, **(E)** Sleep disorders, **(F)** Constipation, **(G)** All-cause mortality.

Figure 3 shows Kaplan-Meier plots in the cohorts, with 1-year follow-up, for each outcome.

During 1-year follow-up, participants with a prescription dispensation of fentanyl, compared to morphine, had a higher risk of fractures, with 1-year cumulative incidence of 6.92 (95%CI of 6.31–7.57) versus 4.13 (95%CI of 3.06–5.39), and falls, with 1-year cumulative incidence of 2.33 (95% CI of 1.99–2.71) versus 1.72 (95%CI of 1.06–2.58) per 1,000 dispensation-month. Conversely, participants with a prescription dispensation of morphine, compared to fentanyl, had a higher risk of cardiac arrhythmia, with 1-year cumulative incidence of 1.97 (95% CI of 1.26–2.88) versus 1.87 (95% CI of 1.56–2.21) and constipation, with 1-year cumulative incidence of 5.97 (95% CI of 4.66–7.48) versus 3.93 (95% CI of 3.47–4.42) per 1,000 dispensation-month compared to the fentanyl cohort.

### 3.2 Association of drug dispensation with adverse outcomes

Table 2 shows the association between adverse events and the prescription dispensations of fentanyl compared to morphine. While on treatment, fentanyl prescription dispensation was significantly associated with a higher risk of fractures compared to morphine [RR of 1.78 (95% CI of 1.25–2.53), HR of 1.63 (95% CI of 1.15–2.32)] with no significant difference observed for the rest of the outcomes. There were insufficient cases of opioid dependence/abuse to perform the analysis.

**TABLE 2:** Risk ratio and hazard ratio for adverse events among incident users of fentanyl prescription dispensations, with morphine incident users as reference.

Adverse events	Risk ratio [95% CI]	Hazard ratio [95% CI]
Cardiac arrhythmia	1.43 [0.81–2.54]	1.45 [0.86–2.44]
Delirium	1.49 [0.7–3.18]	1.3 [0.61–2.77]
Fractures (hip, pelvis, wrist, vertebra, humerus)	1.78 [1.25–2.53]	1.63 [1.15 to 2.32]
Falls	1.54 [0.9–2.63]	1.38 [0.79–2.4]
Sleep disorders (sleep apnea, somnolence)	1.06 [0.55–2.04]	0.88 [0.46–1.68]
Constipation	0.99 [0.71–1.37]	0.84 [0.6–1.16]
All-cause mortality	0.58 [0.24–1.43]	0.55 [0.60–1.16]
Opioid dependence/abuse^a^	-	-

^a^ Insuficient cases were captured in each cohort to analyse Opioid dependence/abuse.

### 3.3 Association of drug dispensations with adverse outcomes stratified by sex and age

The association between the prescription dispensations of fentanyl, compared to morphine, and the adverse outcomes, stratified by sex is reported in the Supplementary Tables S2, S3. Compared to morphine, men who received a prescription dispensation of fentanyl had a higher risk of fractures than women (RR of 3.20 [95% CI of 1.77–5.78] versus 1.48 [95% CI of 0.99–2.23] and HR of 3.16 [95% CI of 1.73–5.77] versus 1.32 [95% CI of 0.88–1.98]). When stratifying by age, an increased risk of fractures was also observed among those younger than 65 years old and those over the age of 80 (RR of 2.67 [95% CI of 1.11–6.45] and 2.07 [95% CI of 1.35–3.18] respectively, HR of 2.60 [95% CI of 1.07–6.28] and 1.68 [95% CI of 1.09–2.60] respectively.

## 4 Discussion

In this retrospective population-based cohort study, compared to morphine users, a higher incidence rate of fractures was observed among fentanyl users. Additionally, the prescription dispensation of fentanyl was significantly associated with greater risks of fractures than the prescription dispensation of morphine. This association was more pronounced in men and among those under the age of 65 and over the age of 80. No significant difference was found in the risks of cardiac arrhythmia, constipation, delirium, falls, opioid dependence/abuse, sleep disorders or all-cause mortality while on treatment.

Morphine and fentanyl are commonly prescribed opioids for managing severe non-cancer pain in primary care (Xie et al., 2022). Head-to-head trials have demonstrated that transdermal fentanyl provides greater pain relief compared to oral morphine, with comparable rates of adverse events (Clark et al., 2004). This contrasts with results from observational studies. For instance, a cohort study utilizing administrative Medicaid data reported a 27% higher risk of emergency department visits associated with fentanyl use compared to morphine (Hartung et al., 2007) and a recent analysis of the FAERS database indicated a higher incidence of adverse drug events among fentanyl users compared to those on morphine (Le et al., 2023).

Although the association between fractures and opioids is well established, prior meta-analyses were hampered by significant variability in effect sizes (Yue et al., 2020; Yoshikawa et al., 2020; Ping et al., 2017), and a lack of adjustment for confounding factors or fracture timing (Yue et al., 2020; Ping et al., 2017).

Our results further analyze this association and corroborates an increased fracture risk among patients who received a prescription dispensation of fentanyl compared to those on morphine in primary care for non-cancer patients. By employing propensity score weighting to adjust for confounders and restricting to on-treatment events, we addressed earlier research limitations and strengthened our findings’ reliability.

The comparative fracture risk between fentanyl and morphine users was also investigated in a 2006 Danish case-control study, which found a higher fracture risk among fentanyl users (OR 2.23) compared to morphine users (OR 1.47) (Vestergaard et al., 2006). However, extrapolating these results to outpatient populations in Spain is challenging due to population baseline risk differences and limited primary care data in the Danish analysis. Our study confirms the increased fracture risk associated with fentanyl use in Spanish outpatient settings. Furthermore, by restricting the analysis to “on-treatment” we have enhanced the robustness of the causal relationship between the current use of fentanyl/morphine and the occurrence of the adverse event.

When stratifying by sex and age, we observed a higher risk of fracture among men compared to women. However, the association between opioids use and increased fracture risk in men has shown inconsistent results.

For example, a prospective cohort study conducted in Finland involving 1,177 men and women, found that the concomitant use of an opioid with an antipsychotic was associated with an increased risk of fracture in men (Nurminen et al., 2012) In contrast, the Mr Os study which included 5,994 community-dwelling men aged 65 years and older, did not identify a significant association between opioid use a fracture risk in this population (Krebs et al., 2016) While this population-based study included 12, 735 men and women with prescription dispensations of morphine or fentanyl which may enhance the generalizability of findings, the absence of adjustment for dose or route of administration limits the interpretation of the results.

The same occurs with age. We found a higher risk of fracture among subjects under 65 and over 80 years old. Given that increasing age is a well-established risk factor for fractures, and that opioid use has also been linked to increased fracture risk, the higher risk of fractures observed in subjects over 80 aligns with existing evidence. The increased risk of fracture on subjects under 65 is counterintuitive and may similarly be attributed to the lack of adjustment by dose of prescribing route.

A higher incidence of falls was observed among patients prescribed fentanyl compared to those on morphine. Conversely, patients using morphine experienced a higher incidence of arrhythmias and constipation compared to fentanyl users. However, no significant associations could be established in any of these cases. Our findings contrast with a prior meta-analysis of 36 cohort and case-control studies, which found a significant association between opioid exposure and falls, fall-related injuries, and fractures (Yoshikawa et al., 2020). One possible explanation for our findings is the underreporting of falls in primary care, as the occurrence of fractures may obscure cases of falls, complicating accurate reporting. While the association between morphine use and arrhythmias has been documented in specific at-risk populations (Sauer et al., 2021; Lee et al., 2016) it still has not been established in primary care patients treated for non-cancer pain. On the other hand, opioid-induced constipation is a well-known adverse effect, affecting up to 60% of individuals who initiate opioid therapy (Coyne et al., 2015) which contrasts with our results. Constipation is a common adverse event of opioids, included in the summary of product characteristics (Electronic Medical Compendium, 2024) which may lead to underreporting in healthcare records as clinicians often assume it to be an expected side effect.

This study has several limitations. First, both fentanyl and morphine are prescribed for managing severe non-cancer pain, including pain after a bone fracture. Although we identified “on-treatment” adverse events, and most incident fractures (88% in the morphine group and 84% in the fentanyl group) occurred at least 30 days after the start of the opioid dispensation, the possibility of reverse causation bias influencing our findings cannot be ruled out. Second, while propensity score weighting balanced observed baseline characteristics between the groups, unmeasured confounders may have biased the estimates. Third, our analysis was based on dispensed packages of fentanyl and morphine, without assessing dose nor the route of administration. Prior studies have shown a dose-dependent relationship between opioid use and fractures (Saunders et al., 2010), which may have influenced the observed associations.

Fourth, this study did not include the analysis of other strong opioids, the cause of death or any analgesic prescription obtained without a prescription, as this information could not be retrieved from the database. Fifth, some outcomes, such as delirium, sleep disorders, falls and constipation may be underreported in routine clinical practice.

Additionally, dispensation of morphine or fentanyl does not necessarily equate to actual drug exposure, however, any non-adherence is likely similar across both groups, potentially minimizing its impact on between-group comparisons. Also, although respiratory depression can occur with the use of morphine or fentanyl, we were unable to capture this outcome, preventing us from estimating its potential association with the study drugs.

At last, although the SIDIAP database was linked with hospitalizations to capture data from inpatients, data from emergency rooms could not be captured which could have led to an underestimation of the fractures that do not require hospitalization.

## 5 Conclusion

In this population-based cohort study, the initiation of a new prescription dispensation for fentanyl, compared to morphine, was significantly linked to an increased risk of fractures. However, these findings should be interpreted cautiously due to the possibility of residual confounding.

## Data Availability

The datasets presented in this article are not readily available because in accordance with current European and national law, the data used in this study is only available for the researchers participating in this study. Thus, we are not allowed to distribute or make publicly available the data to other parties. However, researchers from public institutions can request data from SIDIAP if they comply with certain requirements. Further information is available online (https://sidiap.org/index.php/en/solicituds-en) or by contacting SIDIAP (sidiap@idiapjgol.org). Requests to access the datasets should be directed to Further information is available online (https://sidiap.org/index.php/en/solicituds-en) or by contacting SIDIAP (sidiap@idiapjgol.org).

## References

[B1] Agencia Española de Medicamentos y Productos Sanitarios (2024). Utilización de Medicamentos Analgésicos Opioides en España. Available online at: https://www.aemps.gob.es/medicamentos-de-uso-humano/observatorio-de-uso-de-medicamentos/informes/?lang=gl (Accessed April 15, 2025).

[B2] Alorfi N. M. (2023). Pharmacological methods of pain management: narrative review of medication used. Int. J. Gen. Med. 31 (16), 3247–3256. 10.2147/IJGM.S419239 PMC10402723 37546242

[B3] Clark A. J. Ahmedzai S. H. Allan L. G. Camacho F. Horbay G. L. Richarz U. (2004). Efficacy and safety of transdermal fentanyl and sustained-release oral morphine in patients with cancer and chronic non-cancer pain. Curr. Med. Res. Opin. 20 (9), 1419–1428. 10.1185/030079904X2114 15383190

[B4] Coyne K. S. Margolis M. K. Yeomans K. King F. R. Chavoshi S. Payne K. A. (2015). Opioid-induced constipation among patients with chronic noncancer pain in the United States, Canada, Germany, and the United Kingdom: laxative use, response, and symptom burden over time. Pain Med. 16 (8), 1551–1565. 10.1111/pme.12724 25802051

[B5] Domínguez-Berjón M. F. Borrell C. Cano-Serral G. Esnaola S. Nolasco A. Pasarín M. I. (2008). Constructing a deprivation index based on census data in large Spanish cities (the MEDEA project). Gac. Sanit. 22 (3), 179–187. 10.1157/13123961 18579042

[B6] Drug Abuse Warning Network (DAWN) (2021). Findings from drug-related emergency department visits. Available online at: https://www.samhsa.gov/data/(Accessed December 31, 2024). 27631059

[B7] Electronic Medical Compendium (2024). Morphine Sulphate 10mg/ml solution for injection. Summary of products Characteristics. Available online at: https://www.medicines.org.uk/emc/product/6426/smpc#gref (Accessed December 30, 2024).

[B8] Fleischman R. J. Frazer D. G. Daya M. Jui J. Newgard C. D. (2010). Effectiveness and safety of fentanyl compared with morphine for out-of-hospital analgesia. Prehosp Emerg. Care 14 (2), 167–175. 10.3109/10903120903572301 20199230 PMC2924527

[B9] González-Bermejo D. Rayón-Iglesias P. Rodríguez-Pascual A. Álvarez-Gutiérrez A. Fernández-Dueñas A. Montero-Corominas D. (2021). Drug utilization study on immediate release Fentanyl in Spain. Prevalence, incidence, and indication. Pharmacoepidemiol Drug Saf. 30 (3), 371–378. 10.1002/pds.5118 32929809

[B10] Hartung D. M. Middleton L. Haxby D. G. Koder M. Ketchum K. L. Chou R. (2007). Rates of adverse events of long-acting opioids in a state Medicaid program. Ann. Pharmacother. 41 (6), 921–928. 10.1345/aph.1K066 17504834

[B11] Hu A. M. Shan Z. M. Zhang Z. J. Li H. P. (2021). Comparative efficacy of fentanyl and morphine in patients with or at risk for acute respiratory distress syndrome: a propensity score-matched cohort study. Drugs R. D. 21 (2), 149–155. 10.1007/s40268-021-00338-3 33876394 PMC8054845

[B12] Hurtado I. García-Sempere A. Peiró S. Sanfélix-Gimeno G. (2020). Increasing trends in opioid use from 2010 to 2018 in the region of valencia, Spain: a real-world, population-based study. Front. Pharmacol. 11, 612556. 10.3389/fphar.2020.612556 33362564 PMC7759684

[B13] Krebs E. E. Paudel M. Taylor B. C. Bauer D. C. Fink H. A. Lane N. E. (2016). Association of opioids with falls, fractures, and physical performance among older men with persistent musculoskeletal pain. J. Gen. Intern Med. 31 (5), 463–469. 10.1007/s11606-015-3579-9 26754689 PMC4835377

[B14] Le H. Hong H. Ge W. Francis H. Lyn-Cook B. Hwang Y. T. (2023). A systematic analysis and data mining of opioid-related adverse events submitted to the FAERS database. Exp. Biol. Med. (Maywood) 248 (21), 1944–1951. 10.1177/15353702231211860 38158803 PMC10798186

[B15] Lee C. W. Muo C. H. Liang J. A. Lin M. C. Kao C. H. (2016). Atrial fibrillation is associated with morphine treatment in female breast cancer patients: a retrospective population-based time-dependent cohort study. Med. Baltim. 95 (11), e3102. 10.1097/MD.0000000000003102 PMC4839934 26986153

[B16] Manirakiza A. Irakoze L. Manirakiza S. Bizimana P. (2020). Efficacy and safety of fentanyl compared with morphine among adult patients with cancer: a meta-analysis. East Afr. Health Res. J. 4 (1), 8–16. 10.24248/eahrj.v4i1.617 34308214 PMC8279272

[B17] Nurminen J. Puustinen J. Piirtola M. Vahlberg T. Lyles A. Kivelä S. L. (2012). Opioids, antiepileptic and anticholinergic drugs and the risk of fractures in patients 65 years of age and older: a prospective population-based study. Age Ageing 42 (3), 318–324. 10.1093/ageing/afs178 23204431

[B18] Pierce M. van Amsterdam J. Kalkman G. A. Schellekens A. van den Brink W. (2021). Is Europe facing an opioid crisis like the United States? An analysis of opioid use and related adverse effects in 19 European countries between 2010 and 2018. Eur. Psychiatry 64 (1), e47. 10.1192/j.eurpsy.2021.2219 34165059 PMC8316471

[B19] Ping F. Wang Y. Wang J. Chen J. Zhang W. Zhi H. (2017). Opioids increase hip fracture risk: a meta-analysis. J. Bone Min. Metab. 35 (3), 289–297. 10.1007/s00774-016-0755-x 27023332

[B20] Recalde M. Rodríguez C. Burn E. Far M. García D. Carrere-Molina J. (2022). Data resource profile: the information System for research in primary care (SIDIAP). Int. J. Epidemiol. 51 (6), e324–e336. 10.1093/ije/dyac068 35415748 PMC9749711

[B21] Rosner B. Neicun J. Yang J. C. Roman-Urrestarazu A. (2019). Opioid prescription patterns in Germany and the global opioid epidemic: systematic review of available evidence. PLoS One 14 (8), e0221153. 10.1371/journal.pone.0221153 31461466 PMC6713321

[B22] Sauer F. Jesel L. Marchandot B. Derimay F. Bochaton T. Amaz C. (2021). Life-threatening arrhythmias in anterior ST-segment elevation myocardial infarction patients treated by percutaneous coronary intervention: adverse impact of morphine. Eur. Heart J. Acute Cardiovasc Care 10 (4), 427–436. 10.1093/ehjacc/zuaa005 33620376

[B23] Saunders K. W. Dunn K. M. Merrill J. O. Sullivan M. Weisner C. Braden J. B. (2010). Relationship of opioid use and dosage levels to fractures in older chronic pain patients. J. Gen. Intern Med. 25 (4), 310–315. 10.1007/s11606-009-1218-z 20049546 PMC2842546

[B24] Spencer M. R. Miniño A. M. Warner M. (2022). Drug overdose deaths in the United States, 2001–2021. NCHS Data Brief, no 457. Hyattsville, MD: National Center for Health Statistics. 36598401

[B25] Vestergaard P. Rejnmark L. Mosekilde L. (2006). Fracture risk associated with the use of morphine and opiates. J. Intern Med. 260 (1), 76–87. 10.1111/j.1365-2796.2006.01667.x 16789982

[B26] Xie J. Strauss V. Y. Collins G. S. Khalid S. Delmestri A. Turkiewicz A. (2022). Trends of dispensed opioids in Catalonia, Spain, 2007-19: a population-based cohort study of over 5 million individuals. Front. Pharmacol. 13, 912361. 10.3389/fphar.2022.912361 35754470 PMC9213744

[B27] Yoshikawa A. Ramirez G. Smith M. L. Foster M. Nabil A. K. Jani S. N. (2020). Opioid use and the risk of falls, fall injuries and fractures among older adults: a systematic review and meta-analysis. J. Gerontol. A Biol. Sci. Med. Sci. 75 (10), 1989–1995. 10.1093/gerona/glaa038 32016284

[B28] Yue Q. Ma Y. Teng Y. Zhu Y. Liu H. Xu S. (2020). An updated analysis of opioids increasing the risk of fractures. PLoS One 15 (4), e0220216. 10.1371/journal.pone.0220216 32271762 PMC7145014

