# Supplementary material for: Association of morphine vs. fentanyl prescription dispensation with adverse clinical outcomes
Source: Front Pharmacol. 2025 Jun 25;16:1579634. doi: 10.3389/fphar.2025.1579634 (PMC12237658; doi:10.3389/fphar.2025.1579634)
Supplement: Supplementary file 1 [file DataSheet1.pdf]

Supplementary Material

**Supplementary Table 1.** **ICD 10 codes for the study outcomes**	
**Study outcomes**	**ICD-10 codes**
Delirium	F05, F050, F051, F058, F059, F114, F194
Falls	V00-Y99
Opioid abuse	F11.1
Opioid dependence	F11.2
All-cause mortality	A00-Y89
Cardiac arrhythmia (Atrial fibrillation, flutter, arrythmia, bradyarrhythmia)	A52.06, I48.91, I49.8, I48.92, I49.8, I49.02, I49.9, I49.8
Constipation	K59.00, K59.02, K59.09
Sleep disorders (sleep apnoea, somnolence)	G47.30, R40.0
Fractures	
Osteoporosis with pathological fracture	M80
Fracture of clavicle	S42.0
Fracture of scapula	S42.1
Fracture of humerus	S42.2-S42.4
Fracture of forearm	S52
Fracture of hand	S62.0-S62.4

Fracture of thoracic vertebra	S22.0
Multiple fractures of thoracic spine	S22.1
Fracture of lumbar vertebra	S32.0
Multiple fractures of lumbar spine and pelvis	S32.7
Fracture of other part of pelvis	S32.8
Fracture of spine, level unspecified	T08
Fracture of sternum	S22.2
Fracture of rib	S22.3
Fractures of two or more ribs	S22.4
Flail chest	S22.5
Fracture of sacrum	S32.1
Fracture of coccyx	S32.2
Fracture of ilium	S32.3
Fracture of acetabulum	S32.4
Fracture of pubis	S32.5
Fracture of head and neck of femur	S72.0
Fracture femur	S72.1-S72.4
Multiple fractures of femur	S72.7
Other fractures of femur	S72.8

Fracture of femur, part unspecified	S72.9
Fracture of patella	S82.0
Fracture of tibia	S82.1-S82.8
Fracture of calcaneus	S92.0
Fracture of talus	S92.1
Fracture of other and unspecified tarsal bone(s)	S92.3

Supplementary Table 2. Association between adverse events and fentanyl dispensations, stratified by sex, with morphine as reference.

	Risk Ratio (95% CI)		Hazard Ratio (95% CI)	
	Males	Females	Males	Females
Cardiac arrhythmia	2.09 (0.97-4.52)	1.21 (0.59-2.48)	2.09 (0.95-4.60)	1.23 (0.64-2.33)
Delirium	1.79 (0.47-6.80)	1.39 (0.56-3.44)	1.67 (0.45-6.23)	1.12 (0.46-3.00)
Fractures (hip, pelvis, wrist, vertebra, humerus)	3.20 (1.77-5.78)	1.48 (0.99-2.23)	3.16 (1.73-5.77)	1.32 (0.88-1.98)
Falls	1.55 (0.65-3.70)	1.53 (0.82-2.85)	1.49 (0.62-3.60)	1.32 (0.70-2.52)
Sleep disorders (sleep apnea, somnolence)	0.82 (0.36-1.90)	1.32 (0.46-3.78)	0.77 (0.33-1.80)	1.04 (0.37-2.91)
Constipation	0.74 (0.48-1.12)	1.15 (0.72-1.85)	0.68 (0.44-1.05)	0.94 (0.59-1.51)
All-cause mortality	0.51 (0.12-2.14)	0.64 (0.20- 2.01)	0.51 (0.12-2.15)	0.58 (0.19-1.77)

Opioid dependence /abuse	-	-	-	-

Abbreviations: CI=Confidence Interval. Risk Ratios and Hazard Ratios stated as “-“ means that there are no or too few events to estimate the models.

Supplementary Table 3. Association between adverse events and fentanyl dispensations, stratified by age, with morphine as reference.

	Risk Ratio (95% CI)			Hazard Ratios (95% CI)		
Age	<65	65-80	>80	<65	65-80	>80
Cardiac arrhythmia	-	1.09 [0.39, 3.08]	1.52 [0.78, 2.95]	-	1.85 [0.68, 5.08]	1.23 [0.65, 2.35]
Delirium	-	-	1.45 [0.65, 3.22]	-	-	1.06 [0.48, 2.33]
Fractures (hip, pelvis, wrist, vertebra, humerus)	2.67 [1.11, 6.45]	1.09 [0.55, 2.17]	2.07 [1.35, 3.18]	2.60 [1.07, 6.28]	1.19 [0.61, 2.33]	1.68 [1.09, 2.60]
Falls	0.93 [0.2, 4.37]	0.92 [0.3, 2.88]	1.97 [1.04, 3.75]	0.64 [0.11, 3.73]	1.47 [0.34, 6.41]	1.39 [0.74, 2.64]
Sleep disorders (sleep apnea, somnolence)	0.93 [0.29, 2.96]	2.07 [0.27, 15.93]	0.91 [0.4, 2.08]	0.81 [0.28, 2.37]	2.1 [0.25, 17.68]	0.65 [0.27, 1.55]
Constipation	0.94 [0.34, 2.61]	0.76 [0.41, 1.41]	1.15 [0.79, 1.68]	0.8 [0.28, 2.28]	0.88 [0.49, 1.59]	0.84 [0.55, 1.28]
All-cause mortality	-	-	0.53 [0.2, 1.44]	-	-	0.48 [0.19, 1.25]
Opioid dependence /abuse	-	-	-	-	-	-

Abbreviations: CI=Confidence Interval. Risk Ratios and Hazard Ratios stated as “-“ means that there are no or too few events to estimate the models.
